# Inflammation dynamics modulate periodontal stem cell fate and function

**DOI:** 10.3389/fimmu.2026.1783891

**Published:** 2026-03-05

**Authors:** Tomaz Alves, Carla Alvarez-Rivas, Natcha Mahatumarat, Alpdogan Kantarci

**Affiliations:** 1 Department of Developmental Surgical Sciences, University of Minnesota School of Dentistry, Minneapolis, MN, United States; 2 Oral Biology Department, University at Buffalo, Buffalo, NY, United States

**Keywords:** inflammation, inflammatory resolution, mesenchymal stromal cells, mucosal immunity, periodontal stem cells, pro-resolving mediators, regeneratve microenvironments, single-cell omics

## Abstract

The periodontium hosts diverse populations of mesenchymal stem and progenitor cells that are essential for maintaining homeostasis and driving regeneration. These include cells derived from the periodontal ligament, gingiva, and apical papilla. In health and disease, the fate and function of these stem cell populations are shaped by their microenvironment, particularly by inflammatory signals and their resolution. Chronic inflammation, such as that observed in periodontitis, disrupts the regenerative capabilities, impairing stem cell function and biasing differentiation pathways. Inflammation resolution is an active, instructive process that can restore stem cell plasticity and re-establish regenerative potential. Specialized pro-resolving lipid mediators and immune-regulatory cell types play a central role in this reprogramming. We explore how inflammation and its resolution actively shape the behavior of multiple stem cell compartments in the periodontium, highlight the emerging role of spatially organized immunoregulation, and discuss how these insights may be leveraged to develop regenerative therapies for oral and mucosal tissues. We focused on how inflammatory and resolution signals modulate osteogenic programs in periodontal MSCs and contrast these responses with those in bone marrow–derived MSCs, highlighting source-dependent differences in inflammatory susceptibility and regenerative potential.

## 1 Introduction

The periodontium is a structurally complex system composed of both soft and hard connective tissues that provide anatomical stability and functional support to the tooth (1, 2). The periodontal multi-tissue interface is continuously subjected to mechanical forces and microbial challenges that cause tissue damage. The preservation of periodontal integrity depends on robust regenerative mechanisms sustained by a diverse array of stem and progenitor cell populations residing within the periodontal ligament, gingiva, alveolar bone, and perivascular compartments (3). These cells serve as a reservoir of tissue-forming cells and play a central role in tissue maintenance and adaptation to injury by sensing and responding to immune and environmental cues (4).

Under homeostatic conditions, coordinated interactions between immune cells and mesenchymal stem cells (MSCs) preserve a regenerative microenvironment that supports tissue maintenance and repair (5). This balance is disrupted under prolonged, unresolved inflammatory conditions such as periodontitis, compromising the ability of resident progenitor cells to appropriately sense and respond to microenvironmental stimuli (6). Inflammatory cytokines, microbially derived signals, and oxidative stress act as disruptive cues that reprogram the transcriptional, metabolic, and epigenetic landscape of local stem cells (7). As a result, their self-renewal capacity, lineage specification, immunomodulatory functions, and reparative potential become impaired. Stem cell responses to inflammation are not uniform across the body; their sensitivity and functional outcomes are influenced by tissue origin, niche-specific signals, and prior exposure to inflammatory stimuli (8, 9).

Resolution of inflammation is not a passive dampening of pro-inflammatory signals, but an active, tightly orchestrated biological program. It involves specialized pro-resolving mediators (SPMs), immunoregulatory cell populations, and shifts in cellular metabolism (10). Within the periodontium, these resolution-phase mechanisms have been shown to recondition stem and progenitor cells—restoring regenerative function even in populations previously rendered dysfunctional or senescent by chronic inflammation (11). These insights move beyond the conventional dichotomy of “inflammation versus regeneration,” highlighting a recognized continuum in which resolution serves as a critical phase that directs stem cell fate and tissue repair.

The advent of single-cell and spatial transcriptomics, combined with proteomic and epigenomic profiling, is transforming our understanding of periodontal biology. These approaches have begun to reveal the cellular heterogeneity of stem cell populations in inflamed tissues, their interactions with immune and stromal elements, and the spatial gradients of signaling that govern their behavior. These emerging tools help uncover how prior inflammatory exposures—termed “inflammatory priming”—induce durable changes in their phenotype, secretome, and differentiation behavior, leaving lasting imprints on stem cell function and disease susceptibility.

The focus of this work is to examine how inflammatory and resolution processes influence the fate, plasticity, and function of multiple stem cell populations in the periodontium. We highlight key molecular mechanisms, emerging insights from high-resolution omics, and potential therapeutic strategies that aim to harness these pathways for regenerative dentistry and mucosal immunomodulation.

## 2 Inflammatory cues disrupt homeostatic stem cell niches and alter fate

The periodontium encompasses a diverse array of resident stem and progenitor cell populations—including periodontal ligament stem cells (PDLSCs), epithelial stem cells (ESCs), and gingival mesenchymal stem cells (GMSCs)—that together maintain tissue integrity and drive tissue regeneration in response to injury. These populations reside in a tightly regulated microenvironment where immune signals play instructive roles. However, when the inflammatory response is triggered during gingivitis/periodontitis, broad and often deleterious effects alter the behavior of these cells, shifting their phenotype and modulating their cell fate toward regeneration, dysfunction, senescence, or pathological remodeling.

**Figure 1:**
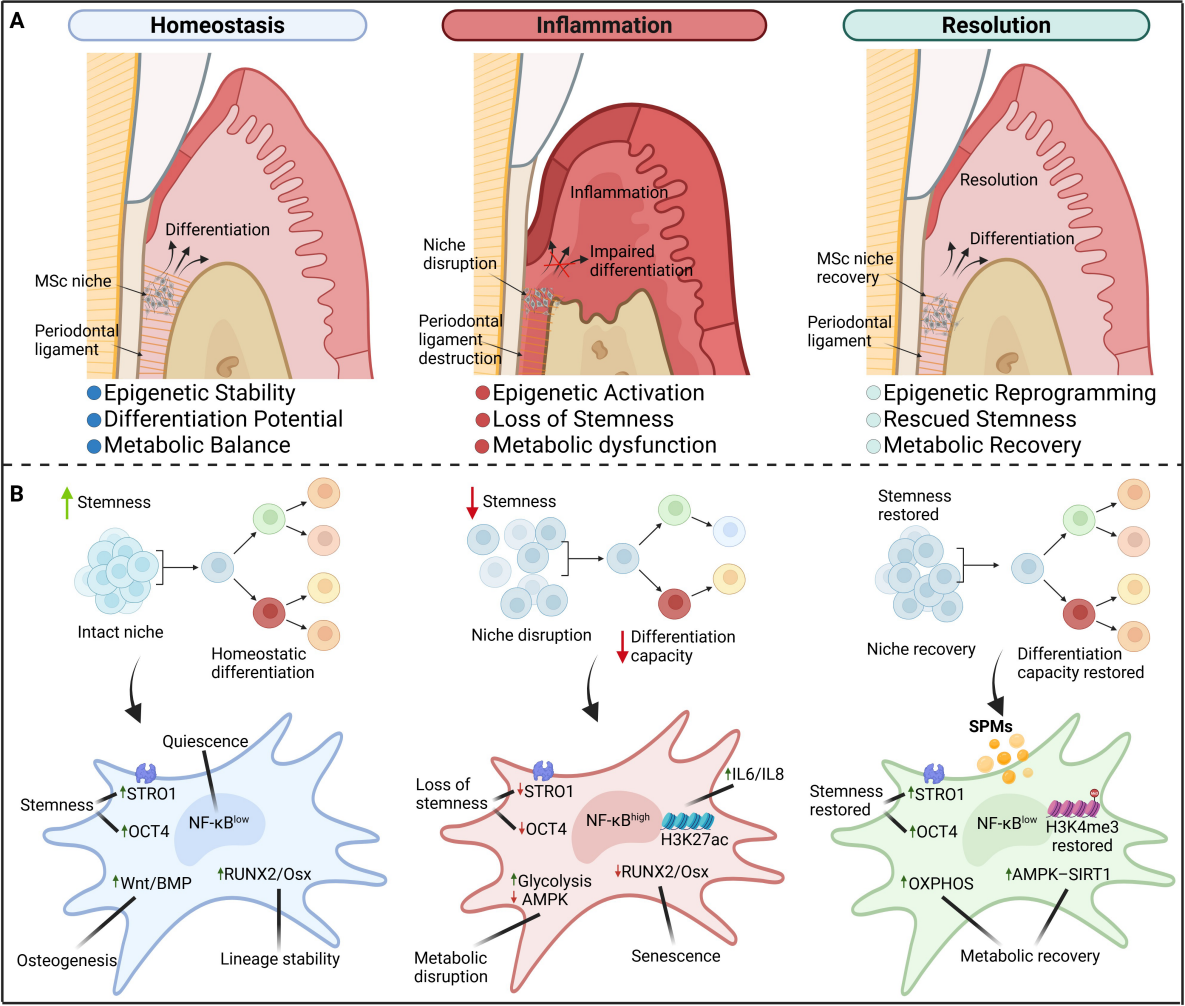
Inflammatory responses and resolution dynamically regulate periodontal stem cell fate and niche function. **(A)** Schematic representation of periodontal stem cell behavior across three immune states. In homeostasis, mesenchymal stem cells **(MSCs)** within the periodontal ligament niche maintain epigenetic stability, metabolic balance, and multilineage differentiation potential, supporting tissue maintenance. During chronic inflammation, sustained inflammatory cues disrupt the stem cell niche promoting periodontal ligament destruction, impair differentiation, and stemness loss, associated with epigenetic activation and metabolic dysfunction. Inflammatory resolution promotes the restoration of the niche, accompanied by stem cell reprogramming, stemness rescue, and recovery of differentiation capacity, enabling tissue repair and regeneration. **(B)** Detailed conceptual model of intrinsic MSC states corresponding to each immune condition. In homeostasis, MSCs remain in a quiescent, stem-like state characterized by high expression of stemness markers (such as STRO1, OCT4), low NF-κB activity, stable chromatin organization, and coordinated Wnt/BMP signaling supporting lineage fidelity and osteogenic potential, with energy production favoring oxidative metabolism. During inflammatory niche disruption, sustained pro-inflammatory signaling induces NF-κB activation, upregulation of inflammatory mediators (e.g., IL-6/IL-8), loss of stemness marker expression, and impaired RUNX2/OSX-dependent differentiation programs. These changes are accompanied by epigenetic remodeling (including increased H3K27 acetylation), metabolic reprogramming toward glycolysis with reduced AMPK activity, and progression toward stem cell dysfunction, exhaustion, or senescence. In the resolution phase, specialized pro-resolving mediators **(SPMs)** promote niche recovery and actively reprogram MSCs by restoring stemness markers, suppressing inflammatory signaling, re-establishing oxidative phosphorylation, and activating AMPK–SIRT1 pathways. This state is associated with epigenetic resetting (including recovery of H3K4me3 patterns), normalized metabolic homeostasis, and reinstatement of multilineage differentiation capacity necessary for periodontal tissue repair and regeneration.

Under homeostatic conditions, resident stem cells are regularly exposed to low levels of microbial challenge; however, this is typically contained through mucosal tolerance and balanced innate immune surveillance (**Figure 1**). During chronic inflammation, as in periodontitis, immune signaling becomes dysregulated, leading to sustained exposure to cytokines such as IL-1β, TNF-α, IL-6, IL-17, and interferons. These signals engage their respective cytokine membrane receptors, as well as microbially derived and damage-associated molecules recognized by pattern recognition receptors (e.g., TLRs and NLRs), which activate downstream pathways (**Figure 1**). Among these pathways are the NF-κB, JAK–STAT, and IRF family transcription factors. While these pathways may acutely enhance stem cell proliferation or mobilization, chronically they promote detrimental outcomes—including stem cell exhaustion, skewed differentiation, loss of quiescence, and impaired regenerative capacity—similar to what has been described in hematopoietic and neural stem cell systems (12).

This inflammatory reprogramming not only alters intrinsic properties of periodontal stem cells—such as self-renewal, survival, and differentiation—but also remodels their surrounding niche. Chronic activation of inflammasome pathways (e.g., NLRP3) and TLRs sustains IL-1 family signalling that drives myeloid-biased ‘emergency’ hematopoiesis and promotes fibrotic marrow remodeling, reducing the pool of multipotent progenitors (13, 14). Concurrently, inflammation perturbs key morphogenetic pathways that guide stem cell fate. For example, Wnt and Notch signaling—critical for maintaining progenitor identity—are downregulated or misdirected in inflamed periodontal niches, shifting differentiation toward less regenerative lineages (15, 16). Similarly, BMP and TGF-β signals, which balance osteogenic and fibrotic programs, become dysregulated, often tipping the balance toward fibrosis (17). Hedgehog, EGF, and FGF signaling—essential for niche organization and stromal support—are also disrupted by inflammation, leading to altered vascular dynamics, ECM composition, and oxygenation (18). These niche−level changes synergize with intracellular transcriptional, metabolic, and signaling alterations to create a feed−forward loop that perpetuates stem cell dysfunction. Together, these insights suggest that in the periodontium, inflammation does not simply suppress regeneration, it fundamentally reshapes the architecture and signaling logic of the stem cell niche.

## 3 Epigenetic and transcriptional rewiring of stem cells within the periodontium

Inflammation exerts long-lasting effects on stem cells by reshaping their transcriptional and epigenetic landscapes (**Figure 1B**) (19). Rather than transient activation of immune-responsive genes, exposure to pro-inflammatory cytokines and microbial ligands can induce stable chromatin remodeling and altered gene expression that persist beyond the initial insult (19). This phenomenon—analogous to “inflammatory priming” described in mesenchymal stromal and myeloid cells—involves promoter CpG demethylation and changes in histone marks such as H3K4me3 and H3K27ac at regulatory loci controlling inflammatory and lineage-associated genes (20). Pro-inflammatory cytokines (IL-1β, TNF-α), chemokines (e.g., CCL2), and growth factors converge on NF-κB, AP-1, and STAT pathways that recruit chromatin-modifying complexes to specific promoters and enhancers, resulting in locus-specific gains of H3K27ac/H3K4me3, increased chromatin accessibility, and reinforced transcription factor binding at inflammatory and lineage-regulatory loci. At inflammatory genes such as CCL2 and IL8, this remodeling manifests as persistently accessible H3K27ac-marked enhancers that facilitate rapid and robust re-induction upon subsequent stimulation, thereby encoding a form of inflammatory “priming” in periodontal stromal cells (21–24).

These epigenetically encoded changes modify the responsiveness of stem and progenitor cells to subsequent stimuli, potentially reinforcing maladaptive immune–stromal feedback loops. Although the precise epigenomic landscape of periodontal mesenchymal stem cells (MSCs) remains incompletely defined, evidence indicates that prior stimulation via pro-inflammatory cytokines (i.e., IL-1β, TNF-α) or PAMPs (TLR2/4 axis) impairs osteogenic differentiation and enhances secretion of pro-inflammatory mediators such as IL-8 (CXCL8) and CCL2 (MCP-1), suggesting osteogenic stem cell-fate impairment (25, 26). In addition, TLR1/4/6 activation suppresses osteogenic gene expression in human PDLSCs through MyD88/TRIF–Akt signaling, while cytokine-induced CCL2 upregulation in PDL fibroblasts recruits additional monocytes and MSCs to the inflamed microenvironment. Collectively, these observations suggest that inflammatory imprinting, while best characterized in bone marrow stromal cells, is a conserved feature of periodontal stromal populations that promotes chronic inflammation and diminishes regenerative potential.

## 4 Lineage bias, stem cell exhaustion, and osteogenic potential

Chronic inflammation imposes sustained stress on resident stem and progenitor cells, leading to maladaptive shifts in their differentiation potential (27). Based on current mechanistic studies, inflammatory lineage bias are more extensive in bone marrow MSCs than in periodontal MSCs. We used bone marrow data as a reference framework and compare them with published work in the periodontium to interpret analogous processes (28–32). In the periodontium, prolonged inflammatory signaling, impairs stemness, reducing their ability to committing to specialized cell types such as osteoblasts, cementoblasts, and fibroblasts (4),. This impairs regenerative competence characteristic of advanced periodontal disease. In bone marrow MSCs, pro-inflammatory cytokines such as TNF-α, IL-1, and IFN-γ disrupt osteogenic transcriptional programs (for example mediated by RUNX2 and osterix) while upregulating fibrotic or adipogenic regulators such as PPARγ and TGF-β. This framework provides a mechanistic pathway for studying how chronic inflammation may skew periodontal MSCs from functional osteogenesis to exhaustion and maladaptive remodeling (30, 33). These effects are further stabilized by downstream activation of signaling axes, including NF-κB and MAPKs, which reprogram stem cell fate (33).

Recent studies indicate that periodontal ligament stem cells and related periodontal MSCs typically exhibit higher proliferative capacity but somewhat lower baseline osteogenic output than bone marrow MSCs. Their osteogenesis is readily suppressed by inflammatory mediators such as TNF-α, which can even enhance osteogenic markers in bone marrow MSCs under similar conditions (28–30). These source-dependent differences suggest that periodontal MSCs may be particularly vulnerable to inflammatory lineage skewing and exhaustion, reinforcing the need to consider tissue origin when extrapolating findings from bone marrow MSCs to periodontal niches (28, 30, 31). Chronic inflammation promotes premature senescence or functional exhaustion in stem cell subsets, particularly under prolonged exposure to oxidative stress and cytokine signaling. Transcriptomic profiling of inflamed periodontal tissues shows upregulation of senescence-associated secretory phenotype (SASP) factors, including IL-6, IL-8, and matrix metalloproteinases (34). Critically, senescent cells lose their proliferative and differentiation capacity and may further sustain local inflammation through paracrine signaling (35). Moreover, metabolic reprogramming—characterized by increased glycolytic flux and mitochondrial dysfunction—reinforces this exhausted state. For example, during active inflammation resolution, specialized pro−resolving mediators such as Maresin−1 have been shown to activate AMPK−linked metabolic programs, improve mitochondrial function, and attenuate NF−κB–dependent inflammatory signalling, changes that are consistent with a shift toward enhanced oxidative phosphorylation and engagement of AMPK–SIRT1–type pathways (36–38). Even after the removal of inflammatory triggers, these imprinted alterations may persist, limiting the effectiveness of regenerative therapies. Together, these findings inform the need for therapeutic strategies that not only resolve inflammation but also reverse senescence-associated reprogramming in periodontal stem cell populations.

## 5 Resolution of inflammation and reprogramming of periodontal stem cells

The Inflammatory-associated reprogramming of stem cells is reversible. During the resolution phase of inflammation, specialized pro-resolving mediators (SPMs)—including resolving (RvE1, RvD1) and maresins (MaR1)—can partially reverse inflammation-induced epigenetic alterations (4). In bone marrow MSCs, these signals modulate chromatin regulators such as histone deacetylases (HDACs) and ten-eleven translocation (TET) enzymes, restoring accessibility at the promoters of osteogenic and matrix-regulatory genes (39).

Epigenetic mechanisms play a pivotal role in restoring stem cell function during inflammatory resolution (19, 40). Inflammation-induced enrichment of the repressive histone mark H3K27me3 at osteogenic and matrix gene promoters, such as RUNX2 and COL1A1, limits differentiation in PDLSCs, and relief of this epigenetic brake—via demethylase activity and pro-resolving signaling—can restore regenerative programs (41). Specialized pro-resolving mediators (SPMs) such as resolvin E1 (RvE1) and maresin 1 (MaR1) have been shown to enhance viability, migration, and osteo/cementogenic differentiation of PDLSCs under inflammatory conditions, suggesting that they may act in part by normalizing chromatin states and transcriptional accessibility at key osteogenic loci (4, 42). Also, resolution is associated with metabolic recovery: inflammation drives a glycolytic, mitochondrial-dysfunctional phenotype, whereas SPMs promote oxidative metabolism, autophagy, and improved bioenergetic balance. In periodontal stromal cells, MaR1 enhances survival and reparative capacity through signaling pathways involving GSK-3β/β-catenin and related metabolic regulators, while activation of pathways such as AMPK, PGC-1α, and SIRT1 in other mesenchymal systems likely contributes to similar effects. Together, these observations underscore that both epigenetic plasticity and metabolic flexibility are central features of stem cell reprogramming and are critical for restoring regenerative competence in inflamed periodontal niches (43–45).

Among the most well-characterized SPMs in the context of the periodontium are resolvins (e.g., RvD1, RvE1), lipoxins (e.g., LXA_4_), protectins, and maresins (e.g., MaR1), which exert their effects via specific G protein-coupled receptors such as ALX/FPR2, ChemR23, and GPR32—many of which are expressed by periodontal ligament stem cells, immune cells, and endothelial components (46–48). Upon engagement, these receptors initiate signaling pathways that attenuate NF-κB activation, suppress the expression of inflammasome components, and promote the expression of anti-inflammatory cytokines such as IL-10 and TGF-β (49). Critically, specialized pro-resolving mediators (SPMs) have been shown to act directly on mesenchymal stromal cells (MSCs), attenuating inflammatory phenotypes and restoring regenerative capacity under inflammatory conditions. In human PDLSCs exposed to LPS, TNF-α, or IL-1β, SPMs such as resolvin E1 (RvE1), lipoxin A_4_ (LXA_4_), and Maresin 1 (MaR1) enhance osteogenic and cementogenic differentiation, in part by increasing the expression of osteogenic regulators, including RUNX2 and BMP2, and by concurrently suppressing pro-inflammatory mediators (50, 51). These effects collectively indicate a re-balancing of differentiation and immune-modulatory gene programs within inflamed progenitor populations. In parallel, emerging evidence suggests that SPMs contribute to metabolic restoration: MaR1 and related mediators improve mitochondrial function, reduce oxidative stress, and promote the survival and reparative capacity of periodontal ligament cells under inflammatory challenge (52). Although the precise mechanisms linking SPM signaling to mitochondrial metabolism in periodontal stem cells remain to be fully defined, these observations underscore that the resolution phase represents more than passive inflammation withdrawal—it is an active and therapeutically exploitable window during which stem cells can be functionally rescued and reprogrammed toward tissue repair.

## 6 Restoration of stem cell niches

Resolution requires re-establishing a functional periodontal stem cell niche. This highly organized microenvironment integrates vascular, neural, immune, and stromal compartments to regulate the survival, quiescence, and differentiation of resident progenitors, such as PDLSCs and gingival MSCs (53). Resolution of inflammation restores the architecture and signaling balance of these niches in part by recruiting and stabilizing immunoregulatory cell types—most prominently FOXP3^+^ regulatory T cells and pro−resolving, M2−like macrophages, with emerging evidence for contributions from other regulatory lymphocyte subsets. These cells secrete anti−inflammatory cytokines, including IL−10 and TGF−β, as well as trophic mediators and growth factors that support extracellular matrix remodeling, epithelial barrier integrity, neurovascular stabilization, and angiogenesis, thereby creating a pro−regenerative milieu(54–57).

Within this context, the spatial proximity of immune, stromal, and endothelial cells to stem and progenitor cells enables direct communication through cell–cell contacts and soluble mediators, re−establishing appropriate cues for polarity, quiescence, or proliferation according to local regenerative demands. Vascular and perivascular domains in particular re−assume their role as regulatory hubs: endothelial and pericyte−derived signals help normalize oxygen tension and metabolite gradients, supply angiocrine and chemokine cues, and condition the metabolic and transcriptional state of neighboring stem cells to favor orderly periodontal repair rather than chronic remodeling (55, 56, 58, 59). Importantly, restoration of the niche is not a passive reset but an active reorganization process that engages both extrinsic and intrinsic components of the periodontal microenvironment. At the intracellular level, this recalibration involves attenuation of inflammatory signaling cascades (for example, reduced activation of NF−κB and pro-inflammatory JAK–STAT axes), normalization of osteo/cementogenic transcriptional programs including RUNX2 and related regulators, and a shift in metabolism away from inflammation-associated glycolysis toward more balanced oxidative phosphorylation and mitochondrial function (4, 33, 56, 60).

Mesenchymal progenitors adopt transcriptional profiles that can temporarily couple immunomodulatory and regenerative roles: inflamed but resolving stromal cells decrease expression of catabolic mediators (such as IL−6, IL−8, and matrix-degrading enzymes) while increasing anti-inflammatory and trophic factors that support matrix remodeling, vascular stability, and epithelial repair. This suggests a feedback mechanism in which stem/progenitor cells help shape the immune milieu while participating in tissue reconstruction. The stability and heterogeneity of these “hybrid” states in periodontal niches remain active areas of investigation rather than fully defined cell states (54, 56).​ Successful resolution thus restores both the intrinsic regenerative capacity of periodontal progenitors and the niche’s ability to coordinate repair across ligament, bone, cementum, and epithelial compartments (57–59).

## 7 Discussion

In this review, we integrated recent evidence on how chronic inflammation and its resolution reshape osteogenic programs in periodontal MSCs and contrasted these findings with the better-characterized inflammatory responses of bone marrow MSCs. It is important to note that the capacity for regeneration within the periodontium is tightly coupled to the immune landscape, which undergoes dynamic changes during inflammation and resolution. Stem and progenitor populations, such as PDLSCs and gingival MSCs, exhibit substantial intrinsic plasticity, but the intensity, quality, and duration of immune signaling profoundly shape their fate and function. Chronic inflammation in periodontitis exposes these cells to persistent pro-inflammatory cytokines, bacterial products, and oxidative stress, which, cumulatively, skew lineage decisions, promote senescence, and suppress regenerative competence. Emerging data is challenging the notion that these alterations are permanently fixed, suggesting instead that at least part of the inflammatory reprogramming of mesenchymal progenitors is reversible in presence of appropriate pro−resolving cues (33, 54, 55, 57, 60).

Resolution−phase signals, particularly lipid−derived SPMs, have emerged as active drivers of stem cell recovery and niche restoration rather than mere terminators of inflammation. In experimental models, SPMs such as RvE1 and MaR1 act directly on periodontal ligament stem/progenitor cells under inflammatory conditions, enhancing their survival, migratory capacity, and osteogenic/cementogenic differentiation while dampening pro−inflammatory gene expression. These effects are accompanied by reactivation of osteogenic transcriptional programs (for example, increased RUNX2 and BMP−related signaling), partial normalization of metabolic status away from highly glycolytic, stress−associated states, and relief of epigenetic constraints on lineage genes in related MSC systems. While detailed *in vivo* maps of transcriptional, metabolic, and epigenetic rewiring during periodontal resolution remain limited, the ability of inflamed progenitors to recover osteogenic or fibroblastic function upon SPM treatment supports the concept of functional “immunoreversibility,” at least in part (37, 48). Translationally, although SPMs show robust proresolving and proregenerative actions in experimental periodontitis, their use in clinical dentistry is currently constrained by limited human trial data, nonstandardized formulations and delivery systems, and uncertainty about long-term efficacy and safety (61).

The dialogue between immune cells and stem/progenitor cells is central to this reparative shift. Macrophage polarization from pro−inflammatory M1−like states toward pro−resolving, M2−like states is a key bridge linking inflammation resolution to the re−establishment of a regenerative microenvironment. M2−like macrophages and regulatory T cells act as both sensors of tissue status and effectors of repair, secreting anti−inflammatory cytokines (e.g., IL−10, TGF−β), growth factors, and matrix−modifying enzymes that support stem cell viability, dampen destructive inflammation, and promote organized matrix remodeling and angiogenesis. In turn, MSCs and periodontal stromal cells can modulate macrophage and T−cell phenotypes via soluble mediators and cell–cell interactions, establishing a feedback loop in which immune and stromal compartments co−reinforce a pro−resolving, pro−regenerative niche (54–56). Importantly, the effects of inflammatory responses on MSC fate and function are not uniform across MSC types. While MSCs from diverse sources share core immunomodulatory features, comparative studies demonstrate source−dependent differences in cytokine sensitivity, immunoregulatory factor expression, and the extent to which osteogenic or chondrogenic programs are impaired under inflammatory conditions. Thus, extrapolations between bone marrow MSCs, PDLSCs, GMSCs, and ESC/iPSC−derived MSC−like cells should be made cautiously and with explicit acknowledgement of these documented variations (62, 63).

Finally, it is noteworthy that although chronic periodontitis is associated with greater marginal bone loss, higher peri-implantitis incidence, and increased implant failure, these outcomes are primarily attributed to persistent dysbiosis and heightened inflammatory responsiveness (64, 65). Emerging data show epigenetic and microRNA alterations at peri-implant sites that mirror periodontitis and may affect healing and osseointegration (66). However, direct evidence linking specific epigenetic or metabolic reprogramming in periodontal MSCs to implant or graft failure remains limited and requires longitudinal validation.

## References

[1] Jepsen S Caton JG Albandar JM Bissada NF Bouchard P Cortellini P . Periodontal manifestations of systemic diseases and developmental and acquired conditions: Consensus report of workgroup 3 of the 2017 World Workshop on the Classification of Periodontal and Peri-Implant Diseases and Conditions. J Periodontol. (2018) 89 Suppl 1:S237–48. doi: 10.1002/JPER.17-0733, PMID: 29926943

[2] Caton JG Armitage G Berglundh T Chapple IL Jepsen S Kornman KS . A new classification scheme for periodontal and peri-implant diseases and conditions-Introduction and key changes from the 1999 classification. J Periodontol J Clin Periodontol J Periodontol. (2018) 89:1. doi: 10.1002/JPER.18-0157, PMID: 29926489

[3] Yang Y Alves T Miao MZ Wu YC Li G Lou J . Single-cell transcriptomic analysis of dental pulp and periodontal ligament stem cells. J Dent Res. (2024) 103:71–80. doi: 10.1177/00220345231205283, PMID: 37982164 PMC10850875

[4] Albuquerque-Souza E Schulte F Chen T Hardt M Hasturk H Van Dyke TE . Maresin-1 and resolvin E1 promote regenerative properties of periodontal ligament stem cells under inflammatory conditions. Front Immunol. (2020) 11:585530/BIBTEX. doi: 10.3389/FIMMU.2020.585530/BIBTEX 33101318 PMC7546375

[5] Lu D Xu Y Liu Q Zhang Q . Mesenchymal stem cell-macrophage crosstalk and maintenance of inflammatory microenvironment homeostasis. Front Cell Dev Biol. (2021) 9:681171. doi: 10.3389/fcell.2021.681171, PMID: 34249933 PMC8267370

[6] Peng Y Li W Zhang Q . Editorial: Immunomodulation of MSCs in tissue repairing and regeneration. Front Immunol. (2023) 14:1150106. doi: 10.3389/fimmu.2023.1150106, PMID: 36860858 PMC9969131

[7] Li H Zhang Y Du S Shen J Liu X Jing J . Remodeling the intestinal immune microenvironment”: immune regulation and tissue regeneration by mesenchymal stem/stromal cells in the repair microenvironment of inflammatory bowel disease. Front Immunol. (2025) 16:1543702. doi: 10.3389/fimmu.2025.1543702, PMID: 40433382 PMC12106535

[8] Choi J Chudziak J Lee J-H . Bi-directional regulation between inflammation and stem cells in the respiratory tract. J Cell Sci. (2024) 137:jcs263413. doi: 10.1242/jcs.263413, PMID: 39508347 PMC11574357

[9] Ho NP-Y Takizawa H . Inflammation regulates haematopoietic stem cells and their niche. Int J Mol Sci. (2022) 23:1125. doi: 10.3390/ijms23031125, PMID: 35163048 PMC8835214

[10] Gilroy DW . Resolving inflammation. Nat Rev Immunol. (2021) 21:620–1. doi: 10.1038/s41577-021-00597-w, PMID: 34580454

[11] Van Dyke TE Sima C . Understanding resolution of inflammation in periodontal diseases: Is chronic inflammatory periodontitis a failure to resolve? Periodontology. (2020) 82:205–13. doi: 10.1111/prd.12317, PMID: 31850636

[12] Nakagawa MM Chen H Rathinam CV . Constitutive activation of NF-κB pathway in hematopoietic stem cells causes loss of quiescence and deregulated transcription factor networks. Front Cell Dev Biol. (2018) 6:143. doi: 10.3389/fcell.2018.00143, PMID: 30425986 PMC6218573

[13] Nguyen T-H Nguyen H-H-N Nguyen T-D Tran VT-H Nguyen H-A Pham D-V . NLRP3 inflammasome activation contributes to the development of the pro-fibrotic phenotype of lung fibroblasts. Biochem Pharmacol. (2024) 229:116496. doi: 10.1016/j.bcp.2024.116496, PMID: 39159876

[14] Pietras EM Mirantes-Barbeito C Fong S Loeffler D Kovtonyuk LV Zhang S . Chronic interleukin-1 exposure drives haematopoietic stem cells towards precocious myeloid differentiation at the expense of self-renewal. Nat Cell Biol. (2016) 18:607–18. doi: 10.1038/ncb3346, PMID: 27111842 PMC4884136

[15] Su Q Huang F Fang X Lin Q . The effect of the Wnt pathway on the osteogenic differentiation of periodontal ligament stem cells in different environments. PeerJ. (2025) 13:e18770. doi: 10.7717/peerj.18770, PMID: 39763707 PMC11702355

[16] Güney Z Kurgan Ş Önder C Tayman MA Günhan Ö Kantarci A . Wnt signaling in periodontitis. Clin Oral Investig. (2023) 27:6801–12. doi: 10.1007/s00784-023-05294-7, PMID: 37814163

[17] Wu M Wu S Chen W Li Y-P . The roles and regulatory mechanisms of TGF-β and BMP signaling in bone and cartilage development, homeostasis and disease. Cell Res. (2024) 34:101–23. doi: 10.1038/s41422-023-00918-9, PMID: 38267638 PMC10837209

[18] Xie Y Su N Yang J Tan Q Huang S Jin M . FGF/FGFR signaling in health and disease. Signal Transduct Target Ther. (2020) 5:181. doi: 10.1038/s41392-020-00222-7, PMID: 32879300 PMC7468161

[19] Zhu X Chen Z Shen W Huang G Sedivy JM Wang H . Inflammation, epigenetics, and metabolism converge to cell senescence and ageing: the regulation and intervention. Sig Transduct Target Ther. (2021) 6:245. doi: 10.1038/s41392-021-00646-9, PMID: 34176928 PMC8236488

[20] Ma J Zhang Y Li J Dang Y Hu D . Regulation of histone H3K27 methylation in inflammation and cancer. Mol BioMed. (2025) 6:14. doi: 10.1186/s43556-025-00254-x, PMID: 40042761 PMC11882493

[21] Larsen SB Cowley CJ Sajjath SM Barrows D Yang Y Carroll TS . Establishment, maintenance, and recall of inflammatory memory. Cell Stem Cell. (2021) 28:1758–1774.e8. doi: 10.1016/j.stem.2021.07.001, PMID: 34320411 PMC8500942

[22] Cao R Thatavarty A King KY . Forged in the fire: Lasting impacts of inflammation on hematopoietic progenitors. Exp Hematol. (2024) 134:104215. doi: 10.1016/j.exphem.2024.104215, PMID: 38580008 PMC11153002

[23] Chen Y Liu S Wu L Liu Y Du J Luo Z . Epigenetic regulation of chemokine (CC-motif) ligand 2 in inflammatory diseases. Cell Prolif. (2023) 56:e13428. doi: 10.1111/cpr.13428, PMID: 36872292 PMC10334270

[24] Walewska A Janucik A Tynecka M Moniuszko M Eljaszewicz A . Mesenchymal stem cells under epigenetic control – the role of epigenetic machinery in fate decision and functional properties. Cell Death Dis. (2023) 14:720. doi: 10.1038/s41419-023-06239-4, PMID: 37932257 PMC10628230

[25] Mao C-Y Wang Y-G Zhang X Zheng X-Y Tang T-T Lu E-Y . Double-edged-sword effect of IL-1beta on the osteogenesis of periodontal ligament stem cells via crosstalk between the NF-kappaB, MAPK and BMP/Smad signaling pathways. Cell Death Dis. (2016) 7:e2296. doi: 10.1038/cddis.2016.204, PMID: 27415426 PMC4973347

[26] Seubbuk S Surarit R Stephens D Hasturk H Van Dyke TE Kantarci A . TLR2 and TLR4 differentially regulate the osteogenic capacity of human periodontal ligament fibroblasts. J Int Acad Periodontol. (2021) 23:3–10. 33512337 PMC8142849

[27] Zhang Z Deng M Hao M Tang J . Stem cell therapy in chronic periodontitis: host limitations and strategies. Front Dent Med. (2022) 2:833033. doi: 10.3389/fdmed.2021.833033, PMID: 41769693

[28] Qu G Li Y Chen L Chen Q Zou D Yang C . Comparison of osteogenic differentiation potential of human dental-derived stem cells isolated from dental pulp, periodontal ligament, dental follicle, and alveolar bone. Stem Cells Int. (2021) 2021:6631905. doi: 10.1155/2021/6631905, PMID: 33927769 PMC8049831

[29] Alves L MaChado V Botelho J Mendes JJ Cabral JMS da Silva CL . Enhanced proliferative and osteogenic potential of periodontal ligament stromal cells. Biomedicines. (2023) 11:1352. doi: 10.3390/biomedicines11051352, PMID: 37239023 PMC10216378

[30] Zhang J Li Z-G Si Y-M Chen B Meng J . The difference on the osteogenic differentiation between periodontal ligament stem cells and bone marrow mesenchymal stem cells under inflammatory microenviroments. Differentiation. (2014) 88:97–105. doi: 10.1016/j.diff.2014.10.001, PMID: 25498523

[31] Kadkhoda Z Motie P Rad MR Mohaghegh S Kouhestani F Motamedian SR . Comparison of Periodontal Ligament Stem Cells with Mesenchymal Stem Cells from Other Sources: A Scoping Systematic Review of *In vitro* and *In vivo* Studies. Curr Stem Cell Res Ther. (2024) 19:497–522. doi: 10.2174/1574888X17666220429123319, PMID: 36397622

[32] Mendoza AH Balzarini D Alves T Rovai ES Holzhausen M . Potential of mesenchymal stem cell sheets on periodontal regeneration: A systematic review of pre-clinical studies. Curr Stem Cell Res Ther. (2023) 18:958–78. doi: 10.2174/1574888X17666220706092520, PMID: 35794765

[33] Zhao Y Shi Y Yang H Liu M Shen L Zhang S . Stem cell microencapsulation maintains stemness in inflammatory microenvironment. Int J Oral Sci. (2022) 14:48. doi: 10.1038/s41368-022-00198-w, PMID: 36216801 PMC9551082

[34] Zhu R Wan H Yang H Song M Chai Y Yu B . The role of senescence-associated secretory phenotype in bone loss. Front Cell Dev Biol. (2022) 10:841612. doi: 10.3389/fcell.2022.841612, PMID: 35223858 PMC8864518

[35] Aquino-Martinez R Eckhardt BA Rowsey JL Fraser DG Khosla S Farr JN . Senescent cells exacerbate chronic inflammation and contribute to periodontal disease progression in old mice. J Periodontol. (2021) 92:1483–95. doi: 10.1002/JPER.20-0529, PMID: 33341947 PMC8281492

[36] Jung TW Kim H-C Abd El-Aty AM Jeong JH . Maresin 1 attenuates NAFLD by suppression of endoplasmic reticulum stress via AMPK–SERCA2b pathway. J Biol Chem. (2018) 293:3981–8. doi: 10.1074/jbc.RA117.000885, PMID: 29414781 PMC5857988

[37] Lovy A Ahumada-Castro U Bustos G Farias P Gonzalez-Billault C Molgó J . Concerted action of AMPK and sirtuin-1 induces mitochondrial fragmentation upon inhibition of ca2+ Transfer to mitochondria. Front Cell Dev Biol. (2020) 8:378. doi: 10.3389/fcell.2020.00378, PMID: 32523953 PMC7261923

[38] Lai J Qian Q Ding Q Zhou L Fu A Du Z . Activation of AMP-activated protein kinase-sirtuin 1 pathway contributes to salvianolic acid A-induced browning of white adipose tissue in high-fat diet fed male mice. Front Pharmacol. (2021) 12:614406. doi: 10.3389/fphar.2021.614406, PMID: 34122060 PMC8193940

[39] Karpenko DV . Inflammatory memory in interleukin-1β promoter methylation of bone marrow-derived stromal cells from healthy humans. Hum Gene. (2025) 43:201367. doi: 10.1016/j.humgen.2024.201367, PMID: 41771284

[40] Bayarsaihan D . Epigenetic mechanisms in inflammation. J Dent Res. (2011) 90:9–17. doi: 10.1177/0022034510378683, PMID: 21178119 PMC3144097

[41] Francis M Pandya M Gopinathan G Lyu H Ma W Foyle D . Histone methylation mechanisms modulate the inflammatory response of periodontal ligament progenitors. Stem Cells Dev. (2019) 28:1015–25. doi: 10.1089/scd.2019.0125, PMID: 31218921 PMC6661920

[42] Alves T Gasparoni LM Balzarini D Albuquerque-Souza E de Oliveira V Rovai ES . Osteogenesis in human periodontal ligament stem cell sheets is enhanced by the protease-activated receptor 1 (PAR1). vivo. Sci Rep 2022 12:1. (2022) 12:1–11. doi: 10.1038/s41598-022-19520-x, PMID: 36117187 PMC9482923

[43] Du L Li Y Liu W . Maresin 1 regulates autophagy and inflammation in human periodontal ligament cells through glycogen synthase kinase–3β/β-catenin pathway under inflammatory conditions. Arch Oral Biol. (2018) 87:242–7. doi: 10.1016/j.archoralbio.2017.12.023, PMID: 29331511

[44] Liu W-C Yang Y-H Wang Y-C Chang W-M Wang C-W . Maresin: macrophage mediator for resolving inflammation and bridging tissue regeneration—A system-based preclinical systematic review. Int J Mol Sci. (2023) 24:11012. doi: 10.3390/ijms241311012, PMID: 37446190 PMC10341548

[45] Jiang H Song D Zhou X Chen F Yu Q Ren L . Maresin1 ameliorates MSU crystal-induced inflammation by upregulating Prdx5 expression. Mol Med. (2023) 29:158. doi: 10.1186/s10020-023-00756-w, PMID: 37996809 PMC10668345

[46] Sahni V Van Dyke TE . Immunomodulation of periodontitis with SPMs. Front Oral Health. (2023) 4:1288722. doi: 10.3389/froh.2023.1288722, PMID: 37927821 PMC10623003

[47] Gireddy HB Rajaram H Koduganti RR Ambati M Harika TSL . Maresins: the mainstay in periodontal resolution. Cureus. (2022) 14(1):e21742. doi: 10.7759/cureus.21742, PMID: 35251814 PMC8888070

[48] Basil MC Levy BD . Specialized pro-resolving mediators: endogenous regulators of infection and inflammation. Nat Rev Immunol. (2016) 16:51–67. doi: 10.1038/nri.2015.4, PMID: 26688348 PMC5242505

[49] Chen Y Wu X Li J Ren Y Miao H Zhai X . The mechanisms of specialized pro-resolving mediators in pain relief: neuro-immune and neuroglial regulations. Front Immunol. (2025) 16:1634724. doi: 10.3389/fimmu.2025.1634724, PMID: 41246324 PMC12611715

[50] Quan H Dai X Liu M Wu C Wang D . Luteolin supports osteogenic differentiation of human periodontal ligament cells. BMC Oral Health. (2019) 19:229. doi: 10.1186/s12903-019-0926-y, PMID: 31655580 PMC6815369

[51] AlZahrani S Shinwari Z Alaiya A Al-Kahtani A . Impact of resolvin-E1 and maresin-1 on bone marrow stem cell osteogenesis under inflammatory stress. Cells. (2024) 13:932. doi: 10.3390/cells13110932, PMID: 38891064 PMC11171860

[52] Yu N Rakian A Dean A Van Dyke TE . Specialized proresolving mediators facilitate the immunomodulation of the periodontal ligament stem cells. Front Dent Med. (2021) 2:701197. doi: 10.3389/fdmed.2021.701197, PMID: 41769693

[53] Zakaria MF Sonoda S Kato H Ma L Uehara N Kyumoto-Nakamura Y . Erythropoietin receptor signal is crucial for periodontal ligament stem cell-based tissue reconstruction in periodontal disease. Sci Rep. (2024) 14:6719. doi: 10.1038/s41598-024-57361-y, PMID: 38509204 PMC10954634

[54] Wang M Xie J Wang C Zhong D Xie L Fang H . Immunomodulatory properties of stem cells in periodontitis: current status and future prospective. Stem Cells Int. (2020) 2020:9836518. doi: 10.1155/2020/9836518, PMID: 32724318 PMC7366217

[55] Chen S Zhou D Liu O Chen H Wang Y Zhou Y . Cellular senescence and periodontitis: mechanisms and therapeutics. Biol (Basel). (2022) 11:1419. doi: 10.3390/biology11101419, PMID: 36290323 PMC9598109

[56] Chen L Zhu S Guo S Tian W . Mechanisms and clinical application potential of mesenchymal stem cells-derived extracellular vesicles in periodontal regeneration. Stem Cell Res Ther. (2023) 14:26. doi: 10.1186/s13287-023-03242-6, PMID: 36782259 PMC9925224

[57] Bharuka T Reche A . Advancements in periodontal regeneration: A comprehensive review of stem cell therapy. Cureus. (2024) 16(2):e54115. doi: 10.7759/cureus.54115, PMID: 38487109 PMC10938178

[58] Hicks MR Pyle AD . The emergence of the stem cell niche. Trends Cell Biol. (2023) 33:112–23. doi: 10.1016/j.tcb.2022.07.003, PMID: 35934562 PMC9868094

[59] Santos AJM Lo Y-H Mah AT Kuo CJ . The intestinal stem cell niche: homeostasis and adaptations. Trends Cell Biol. (2018) 28:1062–78. doi: 10.1016/j.tcb.2018.08.001, PMID: 30195922 PMC6338454

[60] Al Bahrawy M Ghaffar K Gamal A El-Sayed K Iacono V . Effect of inflammation on gingival mesenchymal stem/progenitor cells’ Proliferation and migration through microperforated membranes: an *in vitro* study. Stem Cells Int. (2020) 2020:5373418. doi: 10.1155/2020/5373418, PMID: 32148522 PMC7054781

[61] Hasturk H Schulte F Martins M Sherzai H Floros C Cugini M . Safety and preliminary efficacy of a novel host-modulatory therapy for reducing gingival inflammation. Front Immunol. (2021) 12:704163. doi: 10.3389/fimmu.2021.704163, PMID: 34589083 PMC8475270

[62] Grégoire C Ritacco C Hannon M Seidel L Delens L Belle L . Comparison of mesenchymal stromal cells from different origins for the treatment of graft-vs.-host-disease in a humanized mouse model. Front Immunol. (2019) 10:619. doi: 10.3389/fimmu.2019.00619, PMID: 31001253 PMC6454068

[63] Gugjoo MB Hussain S Shah RA Dhama K . Mesenchymal stem cell-mediated immuno-modulatory and anti-inflammatory mechanisms in immune and allergic disorders. Inflammation Allergy Drug Targets. (2020) 14:3–14. doi: 10.2174/1872213X14666200130100236, PMID: 32000656 PMC7509741

[64] Serroni M Borgnakke WS Romano L Balice G Paolantonio M Saleh MHA . History of periodontitis as a risk factor for implant failure and incidence of peri-implantitis: A systematic review, meta-analysis, and trial sequential analysis of prospective cohort studies. Clin Implant Dent Relat Res. (2024) 26:482–508. doi: 10.1111/cid.13330, PMID: 38720611

[65] Zhang Q Guo S Li Y Li Z Wang D Zhang K . Analysis of risk indicators for implant failure in patients with chronic periodontitis. BMC Oral Health. (2024) 24:1051. doi: 10.1186/s12903-024-04806-5, PMID: 39245715 PMC11382459

[66] Larsson L Giraldo-Osorno PM Garaicoa-Pazmino C Giannobile WV Asa’ad F . DNA and RNA methylation in periodontal and peri-implant diseases. J Dent Res. (2025) 104:131–9. doi: 10.1177/00220345241291533, PMID: 39629934 PMC11752639

